# Integrative Analyses of Metabolome and Transcriptome Reveal Regulatory Network of Puerarin Biosynthesis in *Pueraria montana* var. *lobata*

**DOI:** 10.3390/molecules29235556

**Published:** 2024-11-25

**Authors:** Ting Zhu, Jianing He, Junting Li, Chenxi Liu, Xinyi Min, Xinyi Hu, Xia Liu

**Affiliations:** School of Chemistry, Chemical Engineering and Life Sciences, Wuhan University of Technology, Wuhan 430070, China; sixtnn@163.com (T.Z.); 294016@whut.edu.cn (J.H.); 15294721286@163.com (J.L.); a597879010@outlook.com (C.L.); 17396177935@163.com (X.M.); 18963951698@163.com (X.H.)

**Keywords:** *Pueraria montana* var. *lobata*, puerarin biosynthesis, metabolome, transcriptome, regulatory network

## Abstract

Kudzu, scientifically known as *Pueraria montana* var. *lobata* (Willd.) Maesen & S.M.Almeida ex Sanjappa & Predeep (*P. lobata*), is a perennial vine belonging to the family Leguminosae. Puerarin, a unique constituent and primary active ingredient of this genus, exhibits a broad spectrum of pharmacological activities. This study started with several practical questions: Why is the root the main medicinal part? Why is it not peeled for medicinal purposes? Why is the harvest period usually from December to February? Although the puerarin biosynthesis pathway has been investigated, the stage at which the 8-C glycosylation reaction occurs remains controversial. In this study, metabolomics and transcriptomics analyses were performed on *P. lobata* organs and tissues, including leaves, young stems, mature stems, tuberous cortices, and cortex-excised tubers of roots. Two modules containing genes associated with puerarin biosynthesis were identified by WGCNA. The final selection of important candidate UDP-glucosyltransferases (UGTs) that may be involved in the puerarin biosynthesis pathway included two 8-C-GTs, three 7-O-GTs, and key transcription factors. On this basis, the regulatory network of puerarin biosynthesis was constructed and laid the foundation for the cultivation of high-quality medicinal kudzu with high puerarin content.

## 1. Introduction

Kudzu, *Pueraria montana* var. *lobata* (Willd.) Maesen & S.M.Almeida ex Sanjappa & Predeep (abbreviated as *P. lobata*), is a robust perennial vine in the legume family, with flowering in July–September and fruiting in October–December. It is widely distributed across China, Southeast Asia, and Australia [1,2,3]. Different organs and tissues of *P. lobata* possess distinct pharmacological effects and medicinal value, such as flowers to detoxify alcohol, leaves to make green fertilizer, and stems for papermaking. Kudzu root is an important herbal medicine, and many of its active ingredients are able to exert a wide range of pharmacological activities, such as lowering blood pressure and blood sugar, protecting the liver, detoxifying alcohol, and preventing cardiovascular diseases [4]. *P. lobata* mainly contains pharmacological active substances such as flavonoids, alkaloids, triterpenoids, saponins, coumarins, polysaccharides, etc. [5], in which the flavonoid content is higher than in other active substances, and a variety of flavonoids have been isolated from *P. lobata*, which primarily include puerarin, daidzein, daidzin, formononetin, genistein, and so on. Puerarin is a unique component of this genus and is also the main active ingredient.

In recent years, with the emergence of high-throughput sequencing technology, the genomics, transcriptomics, metabolomics, and proteomics of medicinal plants have been developing rapidly [6], and researchers have gradually reported on the omics of *P. lobata* [7]. However, most studies on the transcriptome of different parts of kudzu do not refer to the genome or are limited to the roots, stems, and leaves, and related metabolomic studies are even fewer; thus, there is a lack of joint analyses of transcription and metabolism.

In the plant body, most of the flavonoids exist in the form of glycosides or carbonyl groups bound to sugars, and some exist in free form. Flavonoids exhibit a wide range of biological activities, such as antioxidant, antibacterial, and antiviral activities, and isoflavones are a significant class of phytoestrogens among flavonoids. Puerarin (8-C-glycoside of daidzein), one of the important isoflavonoids, is a secondary metabolite unique to the Pueraria genus, and research on its biosynthesis has been ongoing. Previous molecular biology studies on legumes, especially soybeans, have provided references for exploring the potential biosynthetic pathways and flavonoid regulatory networks of puerarin [8,9,10].

**Figure 1:**
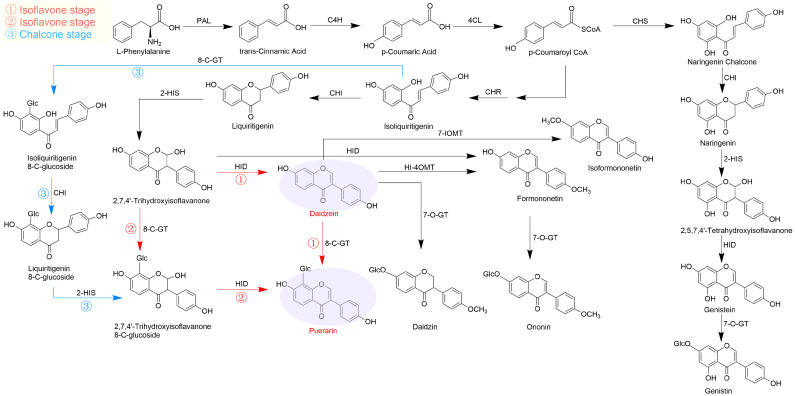
The three possibilities for C-glucosylation in puerarin biosynthesis. Pathways 1 and 2: isoflavone stage; pathway 3: chalcone stage.

**Figure 2:**
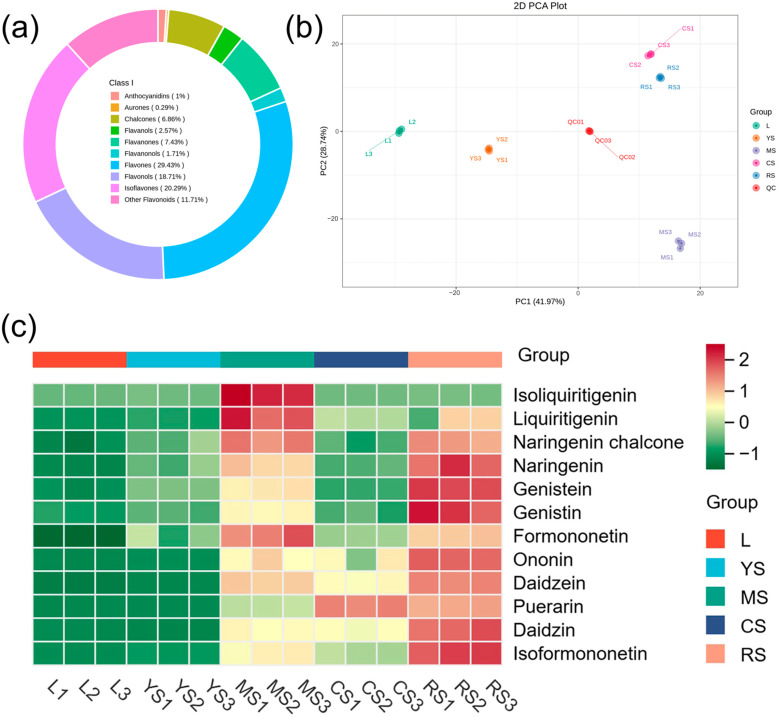
Comprehensive analysis of flavonoid metabolites in *P. lobata*. (**a**) The proportion of flavonoids detected in *P. lobata*. (**b**) PCA of metabolites in different groups. PC1 (first principal component) and PC2 (second principal component) explained 41.97% and 28.74% of the dataset, respectively. (**c**) Heatmap of important flavonoids in puerarin biosynthesis pathway.

Puerarin biosynthesis occurs synergistically through the phenylpropanoid pathway and isoflavone complex pathways. Different flavonoids in plants share a common upstream phenylalanine pathway, which has been well studied. In the downstream isoflavone complex pathway, one of the most important unresolved issues in the biosynthesis of puerarin is the stage at which the 8-C glycosylation reaction occurs; many plant glycosyltransferases have been identified, and several pathways have been hypothesized by researchers to generate puerarin [11]. As shown in Figure 1, 2,7,4′-trihydroxyisoflavanone first generates daidzein under the catalytic action of 2-hydroxyisoflavanone dehydratase (HID) and, later, synthesizes puerarin catalyzed by 8-C-glucosyltransferase (8-C-GT) (Figure 1, pathway 1), or first synthesizes 2,7,4′-trihydroxyisoflavanone 8-C-glucoside under the action of 8-C-GT; this is followed by the synthesis of puerarin in the presence of HID (Figure 1, pathway 2). The most extensively studied pathway is the synthesis of puerarin via the C-glycosylation of daidzein. In addition, the C-glycosyl bond may be formed in the early chalcone stage of the pathway (Figure 1, pathway 3), where isoliquiritigenin is catalyzed by 8-C-GT to produce isoliquiritigenin 8-C-glucoside, followed by the action of CHI to produce liquiritigenin 8-C-glucoside, then the presence of 2-HIS generates the intermediate compounds that are also produced in pathway 2 and finally puerarin as well. However, there are no relevant studies to support this hypothesis. To date, the stage of the C-glycosylation reaction occurring in the puerarin biosynthetic pathway remains controversial [11,12,13].

## 2. Results

### 2.1. Puerarin Content in Root Samples of P. lobata via HPLC

The CS, RS, CD, and RD samples were calculated as dried products, and the content of puerarin is listed in Table 1. The puerarin content in 12 groups of samples were determined separately using high-performance liquid chromatography (HPLC). Comparisons of the samples collected in September and December indicated that the puerarin content was higher in the tuberous cortices than in the cortex-excised tubers of roots. The corresponding chromatograms of the content determination are depicted in Figure S1.

### 2.2. Flavonoid Content and Antioxidant Activity in Different Organs and Tissues of P. lobata

An analysis of Table 2 reveals that the flavonoid content and antioxidant activity of the five organs or tissues followed the orders CS > RS > MS > L > YS for flavonoid content and CS > L > MS > RS > YS for antioxidant activity. Specifically, the tuberous cortices of roots (CS) exhibited the highest flavonoid content and the most potent antioxidant capacity. The leaves (L) demonstrated a high antioxidant capacity despite a relatively low total flavonoid content, while the young stems (YS) showed the lowest flavonoid content and the weakest antioxidant capacity. The standard curves are shown in Figure S2. The sample information is detailed in table of Section 4.1.

### 2.3. Enrichment of Flavonoid Metabolites in Different Organs and Tissues of P. lobata

To elucidate the nutritional and medicinal disparities in various organs and tissues of *P. lobata*, widely targeted metabolite profiling of the samples was conducted. A total of 700 flavonoids were detected, including 7 anthocyanidins, 2 aurones, 48 chalcones, 18 flavonols, 52 flavanones, 12 flavanonols, 206 flavones, 131 flavonols, 142 isoflavones, and 82 other flavonoids. Notably, isoflavones constituted the largest proportion of the flavonoids, accounting for 20.29% (Figure 2a). Comprehensive details regarding these metabolites are presented in Table S1.

Principal component analysis (PCA) was employed to delineate the trend of transcriptome separation between groups, which revealed the presence of transcriptomic differences between sample groups [14]. QC is the quality control sample. The PCA results demonstrated that the distinct organs and tissues of kudzu were completely separated from each other, with significant transcriptomic variations observed between the sample groups (Figure 2b).

The metabolite heatmap (Figure 2c) clearly illustrates that among the numerous flavonoids of *P. lobata*, isoliquiritigenin, liquiritigenin, naringenin chalcone, and formononetin were predominantly accumulated in mature stems, while naringenin, genistein, genistin, ononin, daidzein, puerarin, daidzin, and isoformononetin were more abundant in cortex-excised tubers of roots. Among them, puerarin stood out as the most specific, accumulating predominantly in the tuberous cortices (CS) over the cortex-excised tubers of roots (RS). In contrast, flavonoids in the puerarin biosynthesis pathway were nearly undetectable in leaves and young stems.

### 2.4. P. lobata Transcriptome Analysis Using RNA-Seq

For transcriptomic sequencing, three biological replicates from various organs and tissues of *P. lobata* were utilized, comprising a total of 15 samples. Consequently, 15 cDNA libraries were constructed and sequenced based on Illumina NovaSeq 6000. The average Q30 was 92.82% and the average GC content was 45.00% (Table S2). A total of 615,645,569 reads were localized to the reference genome (Table S3). The comparison efficiency of the transcriptome data with the reference genome ranged from 87.85% to 92.92%, which suggested that the transcriptome data were highly utilized. These findings confirm that the 15 samples in this study were reliable and served as a foundation for subsequent research.

### 2.5. Analysis of DEGs and Functional Annotation

Comparisons between each pair of the five organs and tissues were conducted to derive differentially expressed genes (DEGs) in each pairwise comparison, where the group with the most DEGs was ‘L_vs_RS’, with a total of 13,165 DEGs, including 6758 up-regulated genes and 6407 down-regulated genes. The DEGs in these ten groups were combined and duplicates were removed, yielding a total of 16,528 DEGs. The number of up-regulated and down-regulated DEGs in each group are shown in Table S4 and Figure S3. The FPKM values and annotation information of DEGs are provided in Table S5.

**Figure 3:**
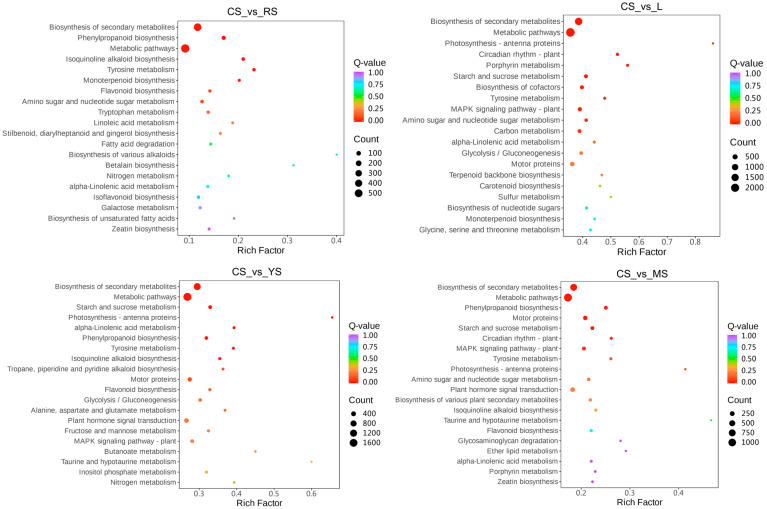
Multi-combination KEGG enrichment scatterplot. The size of the dots represents the number of differential genes enriched in the pathway; the larger the dots are, the more differential genes were enriched in the pathway. The color of the dots represents the significance value of the enriched pathway; the redder the color of the dots, the more significant the enrichment.

The KEGG enrichment results for each comparison combination were sorted by Q-value, and the concatenated set of 15 pathways with the smallest Q-value for each comparison combination was taken for presentation. Since tuberous cortices of roots (CS) were the highest puerarin-containing site, a comparison of ‘CS’ with other groups for differentially expressed genes is presented (Figure 3). The KEGG enrichment analysis revealed that the two most significant pathways were the metabolic pathway (ko01100) and the secondary metabolite biosynthesis pathway (ko01110).

### 2.6. Identification of Structural Genes and Transcription Factors in Puerarin Biosynthetic Pathway

The count of key structural genes involved in the puerarin biosynthesis pathway (Figure 1) was performed according to the method in Section 4.6 (Table S6). Among these genes, 2-HIS and C4H are members of the cytochromeP450 (CYP450) family, both sharing the common Pfam number PF00067, where 2-HIS belongs to the CYP93 subfamily and C4H belongs to the CYP73 subfamily. Subsequently, the genes identified here were differentiated using phylogenetic analyses after intersecting them with module genes by WGCNA. For genes with two Pfam numbers, such as CHS, OMT, and 4CL, the genes with two corresponding Pfam IDs were left in the verification of structural domain integrity and the rest were removed.

Transcription factors (TFs), as a class of proteins that can regulate the transcription process of genes and thus affect gene expression, play an indispensable role in the biosynthesis of puerarin [15]. The screening of key transcription factors was performed according to the method outlined in Section 2.6, and the prediction of transcription factors was facilitated through the iTAK website accessed on 26 May 2024. (http://itak.feilab.net/) [16]. A total of 69 types of transcription factors were identified, with the MYB family accounting for the largest proportion. The results obtained by iTAK are shown in Table S7. According to the existing articles reported [17,18,19,20], this study focused on four types of transcription factors, namely, MYB, bHLH, WRKY, and bZIP. The transcription factors identified by both methods were intersected, yielding 187 MYBs, 159 bHLHs, 105 WRKYs, and 84 bZIPs.

### 2.7. Weighted Gene Co-Expression Network Analysis and Visualization

**Figure 4:**
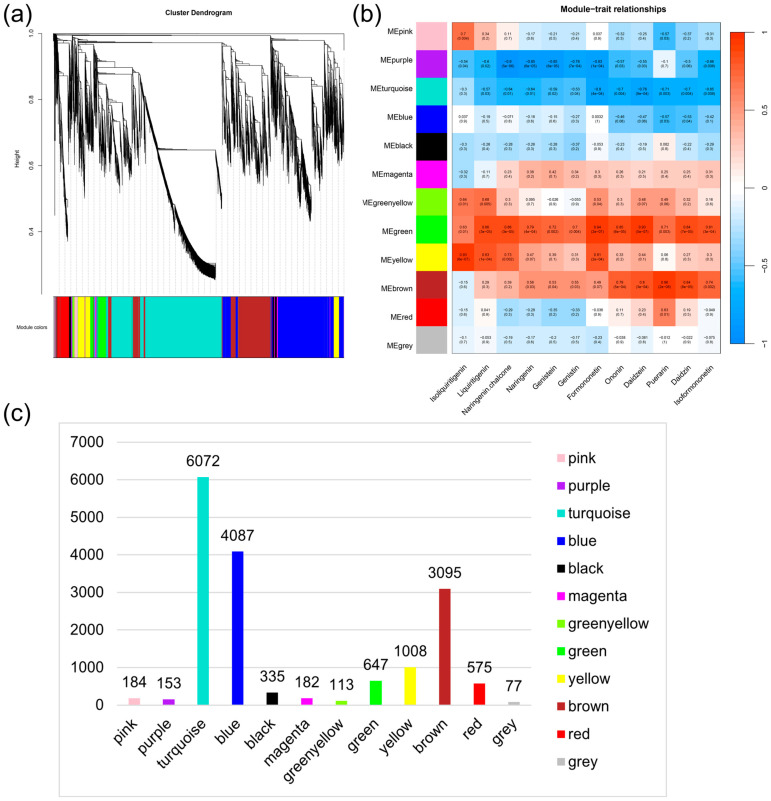
Correlation of genes with flavonoids by WGCNA. (**a**) Gene similarity clustering tree based on topological overlap, with module colors specified after clustering. (**b**) Module gene and metabolite correlation analysis plot. Each row corresponds to a module characterization gene and each column corresponds to a metabolite. Each module contains correlation coefficient and *p*-value. (**c**) Name of each module and number of genes contained within it.

WGCNA, a systems biology approach for characterizing patterns of gene association among samples, enables the identification of highly synergistic gene sets, the discovery of gene sets of interest, and the analysis of significant associations with phenotypes [21,22]. Consequently, we applied WGCNA to construct a network aimed at exploring the relationship between genes like UGTs and important flavonoid metabolites such as puerarin. Characteristic genes, which represent the accumulation of genes contained in each module, were calculated for the genes contained in each module [23], and these genes were clustered into 12 modules, with genes not assigned to any module highlighted in grey (Figure 4). Each co-expression module was assessed for association with flavonoids by the Pearson correlation coefficient.

Correlation analysis between the module-specific characterized genes and key flavonoid metabolites yielded the results presented in Figure 4b. Based on the data from WGCNA, the key genes of the module were identified. By analyzing the correlation between gene modules and important flavonoid metabolites, the genes that characterized different flavonoids can be further identified. As shown in Figure 4b, the brown module was highly correlated with puerarin (r = 0.96) and the green module with daidzein (r = 0.93) and formononetin (r = 0.94) (*p*-value < 0.05). Genes in each module are detailed in Table S8.

The genes of the brown module were intersected with the structural genes derived from the analysis in Section 4.6 to identify the differential structural genes with high correlation in the puerarin biosynthetic pathway, including 2 phenylalanine ammonia lyases (PALs), 1 cinnamate 4-hydroxylase (C4H), 2 4-coumarate-CoA ligases (4CLs), 2 chalcone synthases (CHSs), 1 chalcone reductase (CHR), 1 2-hydroxyisoflavanone synthase (HIS), 7 2-hydroxyisoflavanone dehydratases (HIDs), 5 O-methyltransferases (OMTs), and 37 UDP-glucosyltransferases (UGTs). The genes of the green module, when intersected with the structural genes from Section 4.6, yielded differential structural genes with high correlation in the flavonoid biosynthetic pathway, including two C4Hs, three CHRs, one HID, seven UGTs, which were highly correlated with daidzein, an important precursor of puerarin, and formononetin, a derivative of daidzein. The intersection of the four classes of transcription factors obtained previously (Section 2.6) with the brown module, which was extremely relevant to puerarin, resulted in the identification of 7 MYBs, 24 bHLHs, 12 WRKYs, and 14 bZIPs as candidate key transcription factors for further analysis.

**Figure 5:**
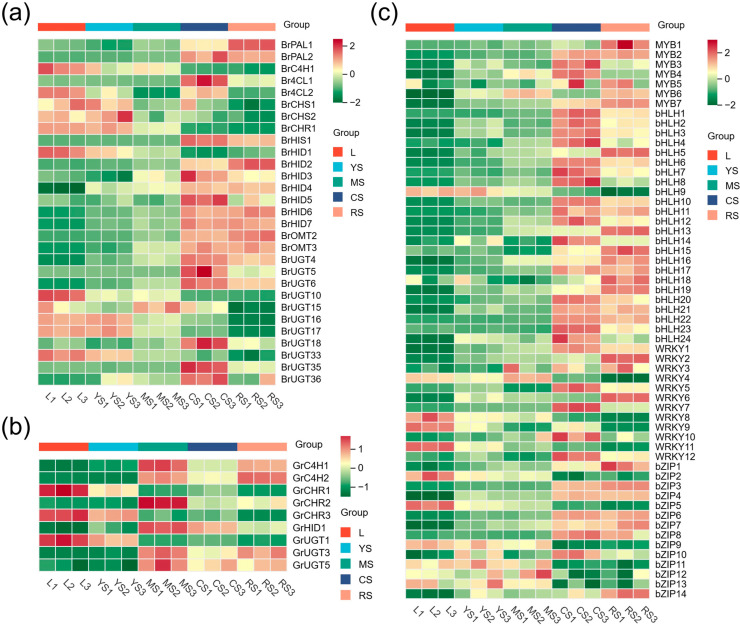
Heatmap of the expression of genes involved in puerarin biosynthesis. Each column represents a group of biological repeats and each row represents a gene. (**a**) Heatmap of expression of key differential structural genes in the brown module. (**b**) Heatmap of expression of differential structural genes in the green module. (**c**) Heatmap of expression of important TFs.

The heatmap results showed a certain regularity in the expression of UGTs. As depicted in Figure 5a, 8-C-GT, which was highly related to puerarin, exhibited a concentrated expression in the tuberous cortices of roots (CS) and cortex-excised tubers of roots (RS), whereas 7-O-GT, which was highly associated with daidzein (Figure 5b), demonstrated accumulation in mature stems (MS) in addition to CS and RS; the majority of transcription factors (TFs) identified through screening that could regulate UGTs were also concentrated in CS and RS. Taken together, HIDs and UGTs, which were implicated in the downstream isoflavone pathway, were almost entirely concentrated in CS and RS, thereby regulating the accumulation and transformation of important isoflavonoids such as puerarin and daidzein. In contrast, genes associated with the upstream phenylalanine pathway, including PAL, C4H, 4CL, etc., were expressed across all parts, although the differences in expression levels were not outstanding.

### 2.8. Phylogenetic Analysis of Candidate UGTs and OMTs

**Figure 6:**
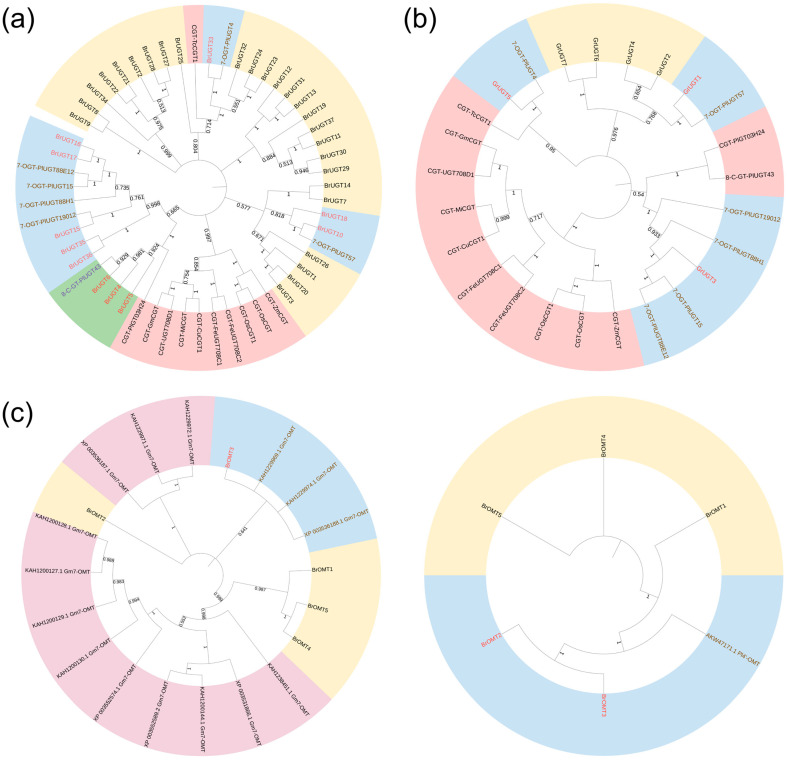
Cluster diagrams for phylogenetic analysis of genes. (**a**) Phylogenetic relationships of UGTs in brown module form *P. lobata* and other plants. (**b**) Phylogenetic relationships of UGTs in green module form *P. lobata* and other plants. (**c**) Phylogenetic relationships of OMTs in brown module form *P. lobata* and other plants.

To further evaluate the potential regulatory roles of candidate UGTs and OMTs, phylogenetic analyses were performed on 37 UGTs and 5 OMTs in the brown module [24,25,26,27], 7 UGTs in the green module, and enzymes with known structures and functions from other species. Genes with homologous sequences and analogous functions typically clustered together on the same branches (Figure 6). The information on the UGTs and OMTs used in the phylogenetic analysis is listed in Table S9.

In the brown module, except for 26 UGTs on the yellow background, which were not assigned to any clade, the remaining 11 UGTs clustered with known UGTs from different species. Among them, eight OGTs clustered with the known 7-O-GTs of *P. lobata*, and three UGTs clustered with the C-GTs. Particularly, three of them (*BrUGT4*, *BrUGT5*, *BrUGT6*) clustered with *PlUGT43*, with *BrUGT6* showing a high degree of homology to *PlUGT43*. *PlUGT43* is recognized as the first 8-C glycosylation that has been proven to directly catalyze daidzein to puerarin during puerarin biosynthesis [28]. Therefore, it is plausible to hypothesize that the three 8-C-GTs identified in this study may share a similar function, which is subject to further verification (Figure 6a). In the green module, three of the seven UGTs (*GrUGT1*, *GrUGT3*, *GrUGT5*) were found to cluster with the 7-O-GTs, while the remaining four did not cluster with the known 7-O-GTs of *P. lobata* (Figure 6b).

Isoflavone 4′-O-methyltransferase (HI-4OMT) and isoflavone 7-O-methyltransferase (7-IOMT) are both classified under isoflavone O-methyltransferase [29], exhibiting functional similarities that make them challenging to differentiate from other candidate enzymes by phylogenetic analysis. Consequently, the candidate OMTs were constructed with known functions of HI-4OMT and 7-IOMT, respectively. In the brown module, it is inferred that *BrOMT2* may function similarly to HI-4OMT, whereas *BrOMT3* may share functional similarities with both HI-4OMT and 7-IOMT (Figure 6c). At the same time, the green module did not have suitable candidate OMTs.

### 2.9. Co-Expression Analysis of Important Genes and Metabolites

**Figure 7:**
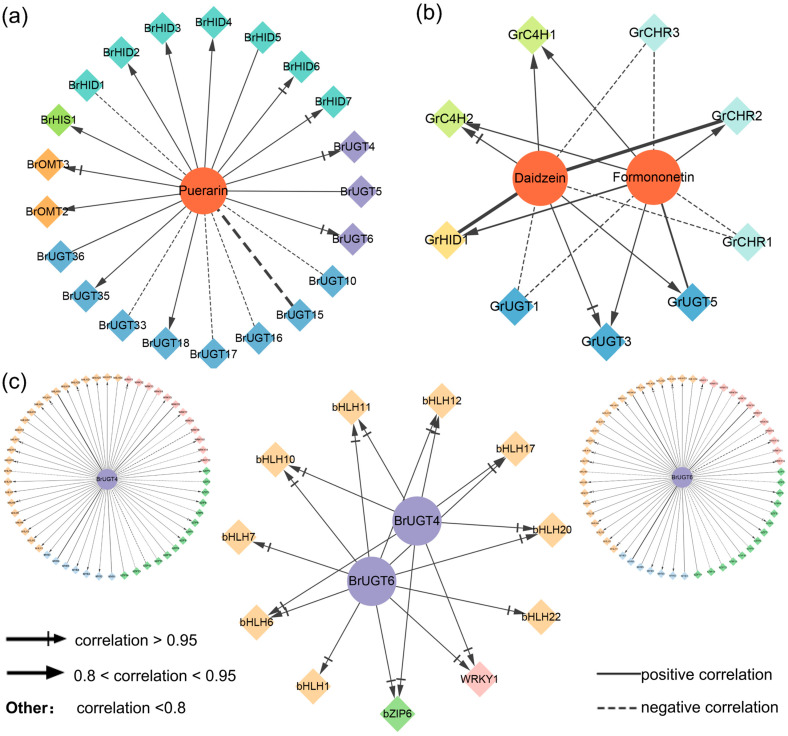
Interaction network maps in puerarin biosynthesis. The thickness of the line represents the size of the *p*-value; a thinner line represents a smaller *p*-value. (**a**,**b**) Interaction network of genes and metabolites involved in puerarin biosynthesis. Circles indicate metabolites and diamonds indicate genes. Different genes are distinguished by different colors. (**c**) Interaction network of TFs and genes involved in puerarin biosynthesis. Circles indicate genes and diamonds indicate TFs.

In order to investigate the relationship between related genes and important metabolites in the puerarin biosynthesis pathway, co-expression network analysis of selected metabolites and genes was performed. UGTs are distinguished by different colors, where 8-C-GT is depicted in purple and 7-O-GT in blue. As shown in Figure 7a, it was observed that *BrOMT3*, *BrHID6*, *BrHID7*, *BrUGT4*, and *BrUGT6* were very strongly correlated with puerarin (correlation coefficient > 0.95), while *BrOMT2*, *BrHID2*, *BrHID3*, *BrHID4*, *BrUGT18*, and *BrUGT35* showed a high correlation with puerarin (0.8 < correlation coefficient < 0.95). *GrC4H2* and *GrUGT3* were highly correlated with both daidzein and formononetin (Figure 7b). The same approach was used to analyze the relevance of transcription factors and candidate genes (Figure 7c), focusing on *BrUGT4* and *BrUGT6*, both 8-C-GT, as potential enzymes capable of catalyzing the generation of puerarin from daidzein. Ultimately, *bHLH1*, *bHLH6*, *bHLH7*, *bHLH10*, *bHLH11*, *bHLH12*, *bHLH17*, *bHLH20*, *bHLH22*, *WRKY1*, and *bZIP6*, which may regulate the expression of *BrUGT4* and *BrUGT6*, were identified.

### 2.10. RT-qPCR Validation of the Puerarin Biosynthesis-Related Genes

**Figure 8:**
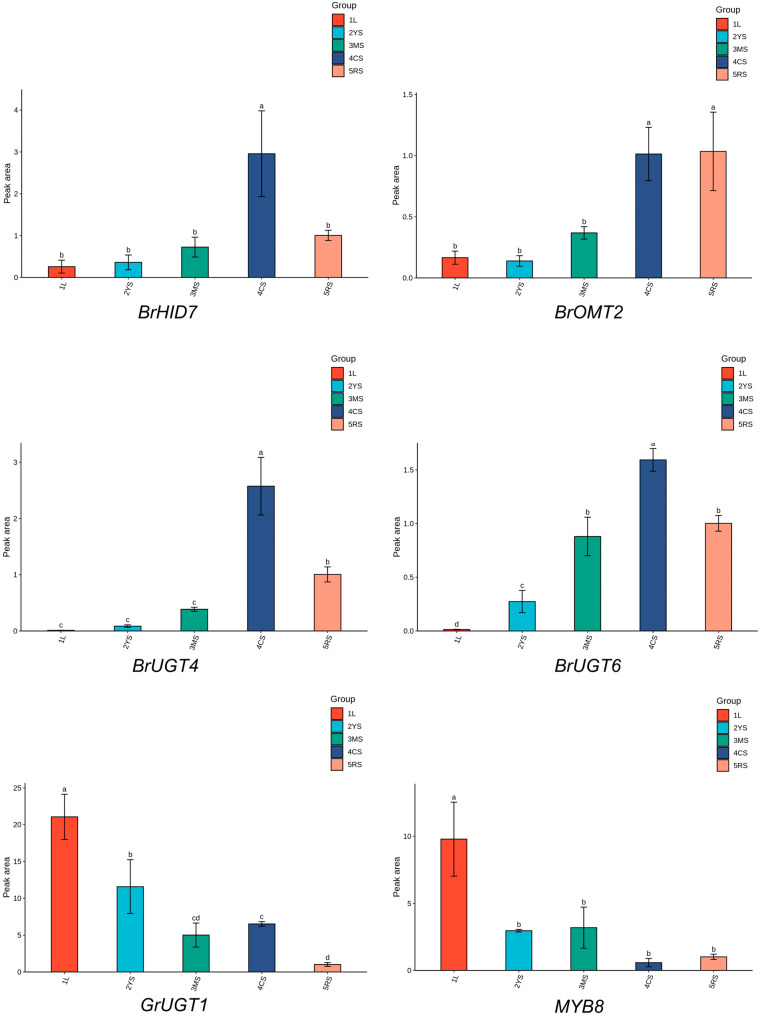
RT-qPCR results of the 6 genes related to puerarin biosynthesis. Analyzed by Duncan’s multiple range test; different letters in the same column indicate significant differences (*p*-value < 0.05, *n* = 3).

To validate the reliability of the RNA sequencing results, the transcript abundance of six genes in each of the five organs and tissues was evaluated using reverse transcription–quantitative polymerase chain reaction (RT-qPCR) [30]. These genes were potentially implicated in the puerarin biosynthesis pathway. The results revealed that the relative expression levels of the genes detected by RT-qPCR were in concordance with the FPKM values derived from the RNA-Seq analysis (Figure 8). This concordance substantiates the reliability of the transcriptomic data in this study, confirming their suitability for further analysis.

## 3. Discussion

In recent years, with the ongoing advancements in transcriptomics and metabolomics technologies, significant progress has been achieved in the study of plant secondary metabolites. To date, researchers have attained a more profound understanding of the puerarin biosynthesis pathway in *Pueraria montana* var. *lobata* [11], identifying key genes and transcription factors that regulate pivotal metabolites. However, most studies on the transcriptomes of different organs and tissues of *P. lobata* do not refer to the genome or are limited to the roots, stems, and leaves, lacking a more detailed dissection. The related metabolomic studies are scarce, leading to a deficiency in the integrated analysis of metabolomes and transcriptomes. Consequently, the process of selecting and breeding high-quality medicinal kudzu varieties is devoid of an enriched theoretical foundation.

Although there is a consensus among researchers regarding the upstream phenylpropanoid pathway in puerarin biosynthesis, the downstream isoflavone pathway remains controversial, particularly concerning the stage at which the 8-C glycosylation reaction occurs. The formation of C-glycosyl flavones involves glycosylation at the 2-hydroxyflavanone intermediate stage and the final product level [31]. Previous researchers have proposed three potential pathways for this problem: (1) daidzein is initially generated under the catalysis of HID, followed by the 8-C-glycosylation of daidzein to obtain puerarin (7,4′-Dihydroxy8-C-glucosylisoflavone); (2) 2,7,4′-trihydroxyisoflavonone undergoes 8-C-glycosylation, with puerarin formation occurring in the presence of HID [11]; and (3) the 8-C glycosylation of isoliquiritigenin at the chalcone stage yields intermediates that eventually produce puerarin [32]. In this study, the metabolomic assay did not detect the four downstream precursor compounds: 2,7,4′-trihydroxyisoflavone, 2,7,4′-trihydroxyisoflavone 8-C-glucoside, isoliquiritigenin 8-Cglucoside, and liquiritigenin 8-C-glucoside. This absence may be attributed to the low concentrations of these precursors, which were below the detection threshold. Thus, the second and third pathways could not be directly substantiated in this study. Nonetheless, large amounts of daidzein were detected in the mature stems, tuberous cortices, and cortex-excised tubers of roots, suggesting that daidzein is most likely to be a substrate for puerarin generation via C-glycosylation, further validating the first pathway for the introduction of 8-C glucoside into daidzein.

In addition, the present study revealed common patterns in the accumulation of metabolites of *P. lobata* in different organs and tissues [33,34,35,36]. Important isoflavonoids in *P. lobata,* such as daidzein, daidzin, and genistein, were mainly concentrated in the cortex-excised tubers of roots, except for puerarin, which was most abundant in the tuberous cortices of roots, followed by the cortex-excised tubers of roots. HPLC determination of the absolute content of puerarin in the tuberous cortices and cortex-excised tubers of roots in September and December instead revealed that the tuberous cortices of roots had a higher content of puerarin, and puerarin content was higher in December than in September. From the point of view of its use as a medicinal herb, this could explain why people choose to harvest kudzu in December–February instead of harvesting it in September when kudzu is in full growth. As a unique component of the Pueraria genus [37], puerarin should not be peeled when used in medicine; otherwise, a lot of puerarin would be lost. In contrast to leaves and young stems, mature stems also contain significant flavonoids, suggesting that in addition to the roots, the mature stems could be used as an alternative part of the kudzu for medicinal purposes and could be harvested before the aboveground parts of the kudzu wither. The cortices of roots were the best in terms of both flavonoid content and antioxidant capacity, reinforcing their medicinal value. Leaves, despite their low flavonoid content, exhibited high antioxidant capacity, indicating the presence of potent antioxidant flavonoids worthy of further investigation. Traditionally, only kudzu roots are used medicinally, neglecting the value of other aboveground parts, and this study can provide a reference for the rational reuse of the nonmedicinal parts of kudzu.

Glycosyltransferases catalyze the attachment of activated sugars to various receptor molecules, and glycosylated products possess numerous biological functions. The UGT family is most intimately associated with the downstream metabolites like puerarin and daidzein among all GT families. The functions of certain *P. lobata* genes, such as PlUGT43, have been confirmed in previous studies, demonstrating their role in catalyzing the production of puerarin from daidzein in soybean hairy roots, where puerarin was not originally present [28]. The analysis of UGTs suggested that three 8-C-GTs may participate in the synthesis of puerarin from daidzein, and three 7-O-GTs may be involved in the synthesis of daidzin from daidzein. Three 8-C-GTs (*BrUGT4*, *BrUGT5*, *BrUGT6*) were closely clustered with *PlUGT43*, with *BrUGT4* and *BrUGT6* showing an extremely strong association with puerarin, suggesting their potential to catalyze the generation of puerarin from daidzein, making these two UGTs the most promising candidate genes. *BrUGT5* was expressed at extremely low levels or nearly absent in all organs and tissues of *P. lobata*, leading to its exclusion from consideration. Three 7-O-GTs (*GrUGT1*, *GrUGT3*, *GrUGT5*) were clustered with *PlUGT57*, *PlUGT15*, and *PlUGT4,* with known functions, respectively, and *GrUGT3* was extremely strongly correlated with daidzein, which suggests that *it* is the most probable candidate gene for 7-O-GT function.

The presence of these candidate genes further substantiates the authenticity of the pathway through which C-glucoside is introduced into daidzein to produce puerarin. However, their functions are not necessarily singular, nor can they be inferred solely from gene sequence and gene expression, and whether they play a role in the biosynthesis of puerarin remains to be further verified.

## 4. Materials and Methods

### 4.1. Plant Materials

**Figure 9:**
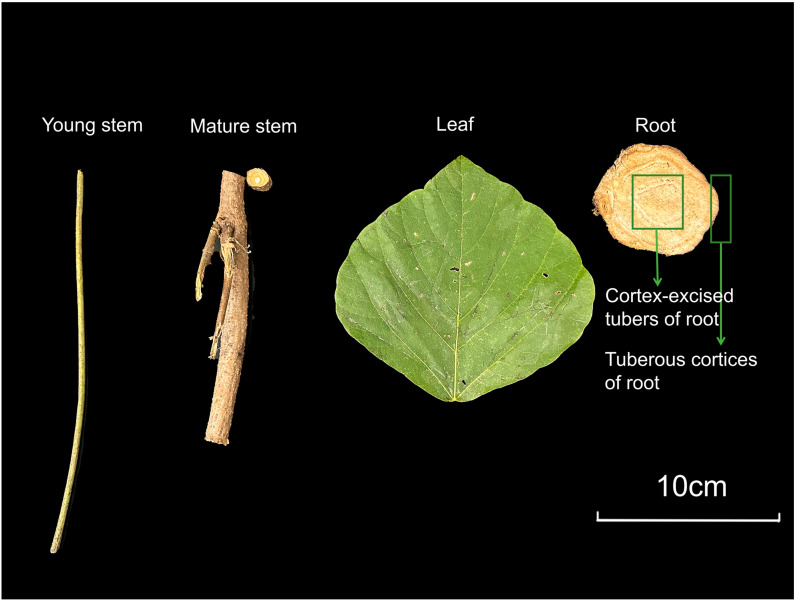
Different organs and tissues of *P. lobata* used in this study.

Different organs and tissues used in this study were harvested from healthy *P. lobata* plants growing in Zhongxiang City, Hubei Province, China (112°87′34′′ E, 31°35′38′′ N, with an altitude of 121.1 m). The fresh root tubers were divided into two parts: tuberous cortices and cortex-excised tubers. All plant materials were cryopreserved at −80 °C for subsequent use. The basic information of the samples is presented in Table 3. Young stem, mature stem, leaf, and root samples were inhibited in the order in Figure 9. All experiments were analyzed with three biological repeats.

### 4.2. Procedures of HPLC to Determine Puerarin

HPLC analysis was performed on a Shimadzu LC-16 system with a Diamonsil C18 column (5 µm C18(2) 250 × 4.6 nm). Methanol–water (25:75) was used as the mobile phase and the detection wavelength was 250 nm. In detail, about 0.1 g of the powder of this product was taken (through the third sieve), precision weighed, and placed in a stoppered conical flask. Then, 50 mL of 30% ethanol was precisely added and the mixture was weighed, reflux-heated for 30 min, cooled, and then weighed, with the 30% ethanol making up for the loss of weight. The mixture then underwent shaking and filtration and, finally, the filtrate was taken as the test product. Puerarin standard (DSTDG000202) was added to 30% ethanol to make a solution of 80 µg/mL, which was used as the control solution. Also, 10 µL of control and test solutions were injected into the liquid chromatograph for subsequent determination [3].

### 4.3. Determination of Flavonoid Content and Antioxidant Activity

The total flavonoids of different organs and tissues of *P. lobata* were extracted as follows: the samples were washed and dried at 60 °C, powdered, and passed through a no. 3 sieve, and the resulting powder was dissolved in 70% ethanol at a solid–liquid ratio of 1:25 (g/mL), condensed, refluxed at 85 °C for 70 min, and filtered to obtain the flavonoid extract [38].

Then, 5.25 mg of puerarin standard was weighed, fixed with 30% ethanol in a 25 mL volumetric flask, and configured into a puerarin standard solution with a concentration of 0.21 mg/mL. Next, 0.2, 0.4, 0.6, 0.8, 1.0, 1.2, 1.4, 1.6, 1.8, 2.0, and 2.2 mL of puerarin standard solution were accurately pipetted into ten 25 mL volumetric flasks and then left for 30 min with shaking, and the UV spectra of puerarin standard solution with a concentration of 0.21 mg/mL were scanned at 200–800 nm; the maximum absorption wavelength was 245.5 nm. The absorbance at 245.5 nm of the diluted puerarin solution with different concentrations was determined using 30% ethanol as a blank control, and the standard curve was plotted. The flavonoid extracts from each part of wild kudzu were diluted with 30% ethanol to the linear range (0.1 mL of the flavonoid extracts were taken and fixed into 25 mL volume), the absorbance at 245.5 nm was determined, and the absorbance value was brought into the regression equation to calculate the concentration. Puerarin was used as the control and 30% ethanol was used as the blank. According to the standard curve of puerarin, the total flavonoid content in the samples was calculated as the number of milligrams of puerarin equivalent per milligram of extract. Flavonoid yield (%) = (C × N × V)/(W × 1000) × 100%.

DPPH radicals and hydroxyl radicals were some of the most important indicators of the antioxidant capacity of the samples. In addition, various antioxidant substances and antioxidant enzymes in the samples constituted the total antioxidant level. Measurement was performed according to the instructions of the DPPH/Hydroxyl Radical Scavenging/Total Antioxidant Capacity T-AOC Assay Kit (Colorimetric Method) of Sangon Biotech Co., Ltd. (Shanghai, China).

### 4.4. UPLC–MS/MS-Based Flavonoid Targeted Metabolomic Analysis

A 1 mg/L solution of 2-chlorophenylalanine was prepared using 70% methanol water as a solvent as an internal standard. Using an electronic balance (MS105DU), 50 mg of sample powder was weighed and 1200 μL of −20 °C pre-cooled internal standard extract was added and vortexed once every 30 min for 30 s for a total of 6 times. After centrifugation at 12,000 rpm for 3 min, the supernatant was aspirated, and the sample was filtered through a microporous filter membrane and stored in an injection bottle for UPLC-MS/MS analysis.

In order to comprehensively determine the flavonoid metabolites of five organs and explore differences in metabolites between samples, the instruments used were UPLC-MS/MS (UPLC, ExionLC™ AD, AB SCIEX, Singapore; MS, QTRAP 6500, AB SCIEX, Singapore); the column was an Agilent SB-C18 (1.8 µm, 2.1 mm × 100 mm). Mobile phase A was ultrapure water containing 0.1% formic acid and mobile phase B was acetonitrile containing 0.1% formic acid. The following program was used (Table 4): the flow rate was 0.35 mL/min, the column temperature was 40 °C, and the injection volume was 2 µL.

Metabolite quantification was accomplished using triple quadrupole mass spectrometry in the multiple reaction monitoring (MRM) mode of analysis, and mass spectrometry data were processed using the software Analyst 1.6.3 [39].

### 4.5. RNA Extraction and RNA Sequencing

The extracted RNA was dissolved in 50 µL of DEPC-treated water using the ethanol precipitation and CTAB-PBIOZOL methods. Subsequently, total RNA was identified and quantified using a Qubit4.0 fluorescence quantifier (Thermo Fisher Scientific, Waltham, MA, USA) and a Qsep400 high-throughput biofragment analyzer (Bioptic Inc., Taiwan, China). After the construction of mRNA libraries, sequencing was performed using the Illumina novaseq 6000. The raw data were filtered using Fastp0.23.2, using Q20 as the threshold for base filtering, and subsequent data analysis was based on clean reads obtained after fine filtering [40,41,42]. Afterwards, the clean reads were compared to the reference genome to obtain mapped data and our study was based on the genomic data of *Pueraria montana* var. *lobata* from the Guangxi Academy of Agricultural Sciences, China, as a reference.

### 4.6. Analysis of Structural Genes in Puerarin Biosynthetic Pathway

The conserved structural domains of the known structural genes in the puerarin biosynthesis pathway were downloaded from the Pfam database accessed on 20 May 2024 (http://pfam.xfam.org/), and the proteins with these structural domains were searched in the protein database of *P. lobata* established in this study using the hmmsearch tool in the HMMER 3.0 software, with a threshold of 1 × 10^−5^. Thus, the protein sequences of the candidate structural genes were obtained.

The protein sequences of related species such as *Pueraria montana* var. *thomsonii* and *Glycine max* were downloaded from NCBI accessed on 20 May 2024 (https://www.ncbi.nlm.nih.gov/), along with homologous comparison of the transcriptome data of *P. lobata* using the local BLAST 2.14.0 software to obtain the desired structural genes.

The protein sequences obtained by the two methods were combined and duplicates were removed. Sequences with incomplete conserved structural domains were manually screened out using the InterPro online protein structure prediction website accessed on 20 May 2024 (https://www.ebi.ac.uk/interpro/) to check the integrity of conserved structural domains.

### 4.7. Analysis of Differentially Expressed Genes in Different Groups

Differential expression analysis between the two groups of the sample was performed on the unstandardized reads data using DESeq2 [43,44], and the false discovery rate (FDR) was obtained by correcting the hypothesis test probability (*p*-value) for multiple hypothesis tests using the Benjamini–Hochberg method [45]. The corrected *p*-value and log2foldchange were used as thresholds for significant differential expression. Differentially expressed genes (DEGs) were screened for |log_2_Fold Change| ≥ 1 and FDR < 0.05.

The Kyoto Encyclopedia of Genes and Genomes (KEGG, https://www.genome.jp/kegg) is a comprehensive database [46] that is used to study the involvement of DEGs in specific biological pathways. After annotating the DEGs in the KEGG database, the enrichment analysis results were graphically displayed using scatterplots.

### 4.8. Weighted Gene Co-Expression Network Analysis

Important metabolites and genes in the puerarin biosynthetic pathway were used for weighted gene co-expression network analysis (WGCNA) [21,22]. Using R software 4.4.0, gene expression and relative metabolite content data were read and examined to remove out-of-specification data. After the test was completed, the samples were clustered according to the gene expression data to test for the presence of outlier samples. The data obtained after the above test were the expression data ready for network analysis. The module and sample correlation data were read and unwanted data were removed. Co-expression modules were identified by R package-weighted gene co-expression network analysis (WGCNA) [23] and visualized on Cytoscape3.10.1. Gene heatmaps were constructed using R4.4.0 programming and software.

### 4.9. Procedures of Real-Time Quantitative Polymerase Chain Reaction

In order to verify the correctness of the structural gene expression profiles, we randomly selected six genes that may be related to the puerarin biosynthetic pathway, using β-actin as an internal reference gene [47]. Specific primers for all the gene candidates to be validated were designed with Primer Premier 5 to ensure an amplification length of 100–150 bp, GC content of 40–60%, and Tm value of 50–60 °C. RT-qPCR primer information is shown in Table S10.

RNA previously extracted and stored at −80 °C was reverse transcribed into cDNA using the ABScript Neo RT Master Mix for qPCR with the gDNA Remover kit (ABclonal Technology Co., Ltd., Wuhan, China). Then, 20 μL of the system was added to the RNase-free PCR tubes on ice, mixed, and centrifuged transiently, followed by the following reaction program on Applied Biosystems 2720 (Thermo Fisher Scientific, Waltham, MA, USA): 37 °C for 2 min, 55 °C for 3 min, 85 °C for 5 min, and, finally, hold at 4 °C to obtain cDNA for each sample. Afterwards, 20 μL of the qPCR reaction system was configured on ice using the BrightCycle Universal SYBR Green qPCR Mix with UDG kit (ABclonal Technology Co., Ltd., Wuhan, China), and the Applied BiosystemsTM QuantStudioTM 3&5 (Thermo Fisher Scientific, Waltham, MA, USA) was used to set up the qPCR reaction program according to the following steps: reaction at 37 °C for 2 min, pre-denaturation at 95 °C for 3 min, and 40 cycles of 95 °C for 5 s, followed by 60 °C for 30~34 s cycles.

All RT-qPCR experiments were divided into three biological replicates; each sample was repeated three times and each reaction was performed in three replicates. The relative expression of each gene was calculated using the 2^−∆∆Ct^ method [48].

## 5. Conclusions

In this study, combined metabolomics and transcriptomics techniques were used to screen and explore important metabolic pathways, metabolites, genes, and transcription factors from a phenotypic and genetics perspective.

Metabolomics data revealed the accumulation pattern of flavonoid metabolites in different organs and tissues of *P. lobata*, with the majority of isoflavonoids in the puerarin biosynthesis pathway concentrated in the cortex-excised tubers of roots, while puerarin accumulated most in the tuberous cortices of roots. Furthermore, the tuberous cortices of roots exhibited the highest total flavonoid content and antioxidant capacity, while the leaves possessed strong antioxidant activity despite their low flavonoid content. These findings offer insights into the selection of medicinal parts of kudzu, highlighting that non-traditional medicinal parts also hold therapeutic potential. Kudzu growing in December has a higher puerarin content than that growing in September, suggesting that a more suitable harvest period for medicinal purposes is December. The mature stems also contain some important flavonoids and can be harvested before the aboveground parts of the kudzu wither to make the best use of them.

The joint analysis of metabolomics and transcriptomics revealed that the brown module with 3095 genes was strongly associated with puerarin and the green module with 647 genes was closely related to daidzein, obtained by WGCNA. Ultimately, two 8-C-GTs that may act on daidzein to generate puerarin and three 7-O-GTs that may act on daidzein to generate daidzin were identified (Table S11). The stages of C-glycosylation occurring in puerarin biosynthesis were elucidated, and the regulatory network of kudzu metabolites, genes, and transcription factors was constructed.

These findings establish a foundation for further investigation into the biosynthesis and regulatory mechanisms of puerarin in *Pueraria montana* var. *lobata*, offering a theoretical basis for subsequent gene function mining and genetic engineering modification. This study will contribute to improving the medicinal value of kudzu, optimize the flavonoids in the whole plant, and provide a reference for the cultivation of new high-quality kudzu varieties.

## Figures and Tables

**Table 1:** Puerarin content of root samples.

Sample	Description	Acquisition Time	Moisture Content (%/Mean ± SD)	Puerarin Content (%/Mean ± SD)
CS	tuberous cortices of roots	September 2023	6.74 ± 0.65 ^b^	3.67 ± 0.36 ^b^
RS	cortex-excised tubers of roots	September 2023	8.33 ± 0.31 ^a^	2.22 ± 0.02 ^c^
CD	tuberous cortices of roots	December 2023	6.50 ± 0.50 ^b^	4.59 ± 0.42 ^a^
RD	cortex-excised tubers of roots	December 2023	9.03 ± 0.33 ^a^	2.41 ± 0.17 ^c^

Analyzed by Duncan’s multiple range test; different letters in the same column indicate significant differences (*p*-value < 0.05, n = 3).

**Table 2:** Flavonoid content and antioxidant activity of samples.

Sample	Flavonoid Content (%/Mean ± SD)	DPPH (%/Mean ± SD)	·OH (%/Mean ± SD)	T-AOC (%/Mean ± SD)
L	2.37 ± 0.31 ^c^	42.39 ± 1.75 ^b^	46.36 ± 7.80 ^ab^	14.9 ± 1.00 ^b^
YS	1.68 ± 0.40 ^c^	21.34 ± 2.13 ^d^	12.74 ± 2.35 ^c^	7.14 ± 0.83 ^d^
MS	5.25 ± 0.89 ^b^	40.43 ± 2.83 ^b^	39.06 ± 4.34 ^b^	11.88 ± 1.43 ^c^
CS	10.75 ± 0.51 ^a^	50.49 ± 0.92 ^a^	59.11 ± 7.37 ^a^	18.90 ± 0.86 ^a^
RS	5.67 ± 0.33 ^b^	32.13 ± 0.32 ^c^	31.99 ± 5.05 ^b^	8.36 ± 0.29 ^d^

Analyzed by Duncan’s multiple range test; different letters in the same column indicate significant differences (*p*-value < 0.05, n = 3).

**Table 3:** Sample information of *Pueraria montana* var. *lobata*.

Sample	Description	Acquisition Time
L	Leaves	September 2023
YS	Young stems	September 2023
MS	Mature stems	September 2023
CS	Tuberous cortices of roots	September 2023
RS	Cortex-excised tubers of roots	September 2023
CD	Tuberous cortices of roots	December 2023
RD	Cortex-excised tubers of roots	December 2023

**Table 4:** UPLC program settings.

Time/min	A (%)	B (%)
0	95	5
9	5	95
10	5	95
11.1	95	5
14	95	5

## Data Availability

The original contributions presented in this study are included in this article/the Supplementary Materials, and further inquiries can be directed to the corresponding author.

## Supplementary Materials

The following supporting information can be downloaded at https://www.mdpi.com/article/10.3390/molecules29235556/s1, Figure S1: The related chromatograms of the content determination; Figure S2: The standard curves; Figure S3: The number of up-regulated and down-regulated DEGs; Table S1: The detailed information of all metabolites; Table S2: Reads information statistics of different groups; Table S3: Reads mapped information statistics of different groups; Table S4: DEG information statistics of different groups; Table S5: DEG FPKM values and annotation information; Table S6: DEGs identified related to puerarin biosynthesis; Table S7: Transcription factor information predicted through the iTAK website; Table S8: Genes in each module by WGCNA; Table S9: The information of UGTs and OMTs used in phylogenetic analysis; Table S10: Primer sequences used for qPCR; Table S11: The protein sequences of candidate UGTs.
