# Supplementary figures and images for: Integrative Analyses of Metabolome and Transcriptome Reveal Regulatory Network of Puerarin Biosynthesis in Pueraria montana var. lobata
Source: Molecules. 2024 Nov 25;29(23):5556. doi: 10.3390/molecules29235556 (PMC11643513; doi:10.3390/molecules29235556)

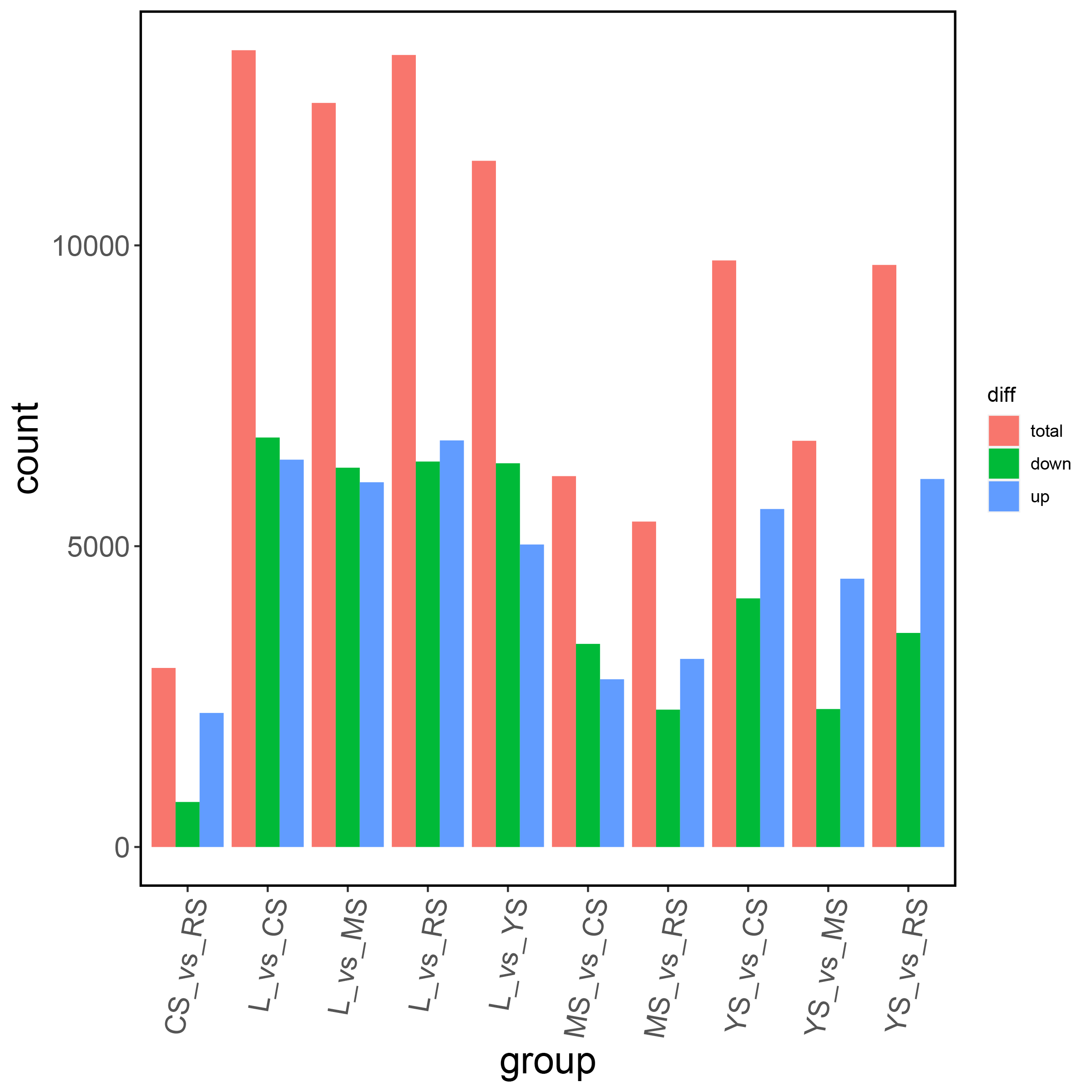

Supplement: Supplementary file 1 [file molecules-29-05556-s001.zip › FigureS3.tif]
